# Don’t Skip the Color: Emergency Department Neonatal Point-of-Care Ultrasound Diagnosis

**DOI:** 10.1016/j.acepjo.2026.100461

**Published:** 2026-06-30

**Authors:** Joseph B. Walsh, Tasha Desai

**Affiliations:** 1 Department of Emergency and Hospital Medicine, Jefferson Health - Lehigh Region, Bethlehem, Pennsylvania, USA; 2 Department of Emergency and Hospital Medicine, Division of Pediatric Emergency Medicine, Jefferson Health - Lehigh Region, Bethlehem, Pennsylvania, USA

**Keywords:** congenital defect, POCUS, pediatrics

## 1 Patient Presentation

**Figure 1:**
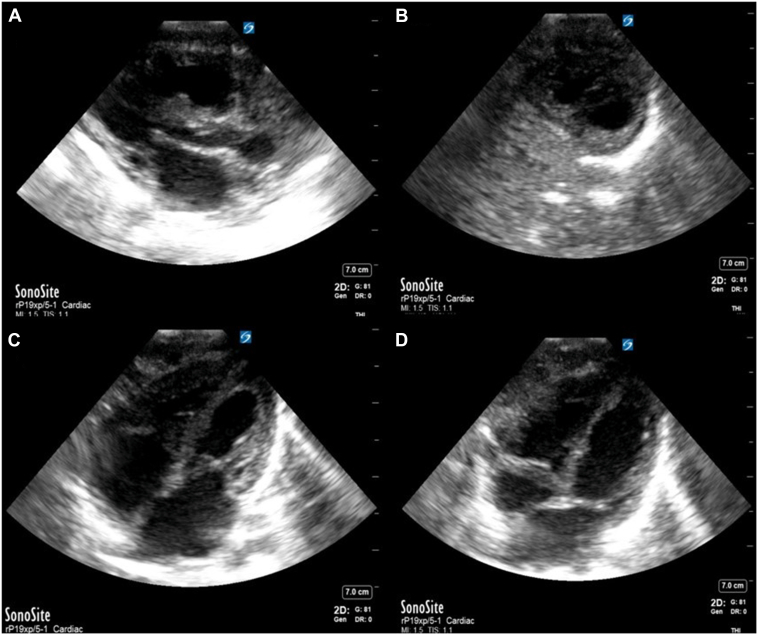
Standard (noncolor flow) images obtained during bedside point-of-care ultrasound echocardiogram performed in the emergency department. No significant abnormalities were noted in the parasternal long axis view (A), parasternal short axis view (B), apical 4-chamber cardiac view (C), or the subxiphoid cardiac view (D) using this imaging modality.

**Figure 2:**
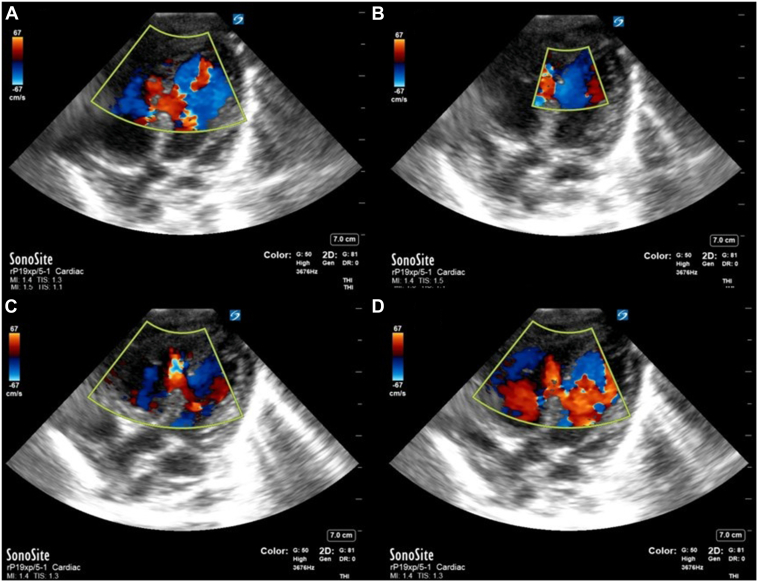
Color flow images obtained during bedside point-of-care ultrasound echocardiogram performed in the emergency department. The addition of color flow allowed for visualization of blood shunting from the left side of the heart to the right side, concerning for ventricular septal defect. Panels (A-D) show snapshots at different times throughout the cardiac cycle from the apical 4-chamber cardiac view.

A 3-week-old full-term male presented with irritability, nasal congestion, cough, decreased feeding, and increased work of breathing. He had a prior neonatal intensive care unit admission for transient tachypnea of the newborn. On examination, he was tachypneic with subcostal retractions, though his oxygen saturation remained normal at 96% on room air. Cardiac examination revealed prominent heart sounds without a clear murmur. Point-of-care ultrasound (POCUS) demonstrated normal cardiac structure on grayscale imaging, though color Doppler revealed left-to-right flow across the interventricular septum (Figs 1 and 2).

## 2 Diagnosis

### 2.1 Congenital Ventricular Septal Defect

Neonates with congenital heart disease often present with nonspecific symptoms, making diagnosis challenging. POCUS is a valuable bedside diagnostic tool but may miss intracardiac shunts when limited to grayscale imaging.^1^

Color Doppler enhances detection of abnormal flow patterns and should be considered in neonates with unexplained tachypnea, as it allows for visualization of abnormal blood flow within the heart. Early identification of congenital cardiac abnormalities allows for timely consultation and management.^2^

Current literature supports expanding use of POCUS in pediatric and neonatal populations, though it should complement, not replace, formal echocardiography.^3^^,^^4^

At 3 months of age, the patient underwent surgical repair complicated by ventricular dysfunction and valvular regurgitation, requiring medical therapy. Despite these complications, the surgery was ultimately successful, and the patient was discharged home with outpatient follow-up scheduled for pediatrics and cardiology.

## Funding and Support

By *JACEP Open* policy, all authors are required to disclose any and all commercial, financial, and other relationships in any way related to the subject of this article as per ICMJE conflict of interest guidelines (see www.icmje.org). The authors have stated that no such relationships exist.

## Conflict of Interest

All authors have affirmed they have no conflicts of interest to declare.
